# Fc receptor‐like A promotes malignant behavior in renal cell carcinoma and correlates with tumor immune infiltration

**DOI:** 10.1002/cam4.70072

**Published:** 2024-08-06

**Authors:** Jun‐peng Liu, Yi‐fan Jiang, Jin‐wen Liu, Chong‐jiang Tian, Yu‐zhao Lin, Yun‐zhi Yang, Ze‐ke Zhang, Yi‐liang Fang, Bin Huang, Hao Lin

**Affiliations:** ^1^ Department of Urology The Second Affiliated Hospital of Shantou University Medical College Shantou China; ^2^ Department of Urology, The First Affiliated Hospital of Sun Yat‐sen University Guangzhou China

**Keywords:** FCRLA, immune infiltration, MMP2, pERK1/2, renal cell carcinoma

## Abstract

**Background:**

Our study aims to investigate the mechanisms through which Fc receptor‐like A (FCRLA) promotes renal cell carcinoma (RCC) and to examine its significance in relation to tumor immune infiltration.

**Materials and Methods:**

The correlation between FCRLA and data clinically related to RCC was explored using The Cancer Genome Atlas (TCGA), then validated using Gene Expression Omnibus (GEO) gene chip data. Enrichment and protein–protein interaction (PPI) network analyses were performed for FCRLA and its co‐expressed genes. FCRLA was knocked down in RCC cell lines to evaluate its impact on biological behavior. Then the potential downstream regulators of FCRLA were determined by western blotting, and rescue experiments were performed for verification. The relevance between FCRLA and various immune cells was analyzed through GSEA, TIMER, and GEPIA tools. TIDE and ESTIMATE algorithms were used to predict the effect of FCRLA in immunotherapy.

**Results:**

Fc receptor‐like A was associated with clinical and T stages and could predict the M stage (AUC = 0.692) and 1–3‐ and 5‐year survival rates (AUC = 0.823, 0.834, and 0.862) of RCC patients. Higher expression of FCLRA predicted an unfavorable overall survival (OS) in TCGA‐RCC and GSE167573 datasets (*p* = 0.03, *p* = 0.04). FCRLA promoted the malignant biological behavior of RCC cells through the pERK1/2/‐MMP2 pathway and was associated with tumor immune microenvironment in RCC.

**Conclusion:**

Fc receptor‐like A is positively correlated with poor outcomes in RCC patients and plays an oncogenic role in RCC through the pERK1/2‐MMP2 pathway. Patients with RCC might benefit from immunotherapy targeting FCRLA.

## 1 INTRODUCTION

Renal cell carcinoma (RCC) is a malignancy arising from the renal tubular epithelial system, which accounts for approximately 90% of renal malignancies and 4.2% of all malignant tumors in adults.^1^ It was estimated that there were about 746,080 new cases of RCC and 13,780 deaths in the USA in 2021.^2^ Moreover, clear cell carcinoma of the kidney (KIRC) is the most common type, accounting for 60%–85% of all RCC cases.^3^ Due to the asymptomatic nature of early‐stage RCC,^4^ most patients are diagnosed at an advanced stage. Even though surgical treatment is performed, 33% of advanced localized RCC patients develop metastasis,^5^ and the survival of these patients was only 10 months, especially worse than others.^6^, ^7^ Therefore, the prognosis of patients with renal cell carcinoma is not optimistic.

Surgical treatment is the primary option for patients with localized RCC. However, the 10‐year cumulative incidence of recurrences after treatment is reported to be 20%–30%.^8^, ^9^ With the keeps developing of nowadays medical, the application of immunotherapy has been gradually used in advanced RCC.^10^ Immune checkpoint inhibitors (ICIs) show promise in achieving durable remission.^11^ While ICI‐based combinations have dramatically improved outcomes for patients with metastatic RCC, most patients still either have primary resistance to these therapies or acquire resistance after an initial response.^12^ Moreover, immune‐related adverse events of the existing ICIs are high, reaching up to 70%.^13^ Given this context, there is an urgent need for reliable diagnostic markers for early‐stage RCC and new immunotherapy targets for advanced RCC.

FCRL family members are involved in regulating damage caused by IgG‐related antigens. The structure of Fc receptor‐like A (FCRLA), also known as FCRL1, resembles that of conventional receptors for the Fc portion of immunoglobulin, which are expressed in the cytosol of B cells.^14^ FCRLA can bind to intracellular immunoglobulins, thereby regulating the processes of immunoglobulin synthesis and secretion.^15^ Based on the function of the B cell‐related gene, FCRLA may predict the efficiency of DC1‐mediated immune checkpoint blockade therapy.^16^ In addition, FCRLA has been shown to participate in immune response‐related pathways in various malignancies, including advanced laryngeal cancer, hepatocellular carcinoma, and ovarian cancer, hepatocellular carcinoma and ovarian cancer.^17^, ^18^, ^19^ However, the mechanism of FCRLA in RCC remains unclear.

Matrix metallopeptidase 2 (MMP2) is a zinc‐related proteinase involved in tumor metastasis.^20^ MMP2 has been reported to be regulated through phosphorylation‐ERK1/2 in gastric cancer.^21^ It is notable that MMP2, which was regulated by several proteins, such as glucose‐6‐phosphate dehydrogenase, is highly expressed in RCC.^22^ Our study aims to investigate the underlying molecular mechanism of FCRLA and MMP2 in RCC development, with the goal of identifying promising diagnostic biomarkers and effective immunotherapeutic targets for RCC treatment.

## 2 MATERIALS AND METHODS

### 2.1 Data sources

The Cancer Genome Atlas (TCGA) (https://cancergenome.nih.gov/) is a comprehensive publicly available database. Eight hundred eighty‐six TCGA‐RCC samples were selected for further analysis. Then the RNA expression of FCRLA was extracted from TCGA‐RCC RNA‐sequence data. Patient clinicopathological data and FCRLA expression were also obtained from the corresponding TCGA‐RCC clinical data.

Selection criteria of the GEO gene chip required that the number of RCC samples must be more than 50, and each sample needed to have corresponding survival data. Then GSE167573 was selected and downloaded from the Gene Expression Omnibus (GEO) database (https://www.ncbinlm.nih.gov/geo/), and the related FCRLA RNA expression and overall survival data were extracted. All data was downloaded from the UCSC Xena browser (https://xenabrowser.net/).^23^

### 2.2 Analysis of overall survival (OS) and clinicopathologic data, and construction of receiver operating characteristic (ROC) curves

Kaplan–Meier analysis with the log‐rank test was performed to depict the OS curves. ROC curves were used for measuring the predictive efficacy of FCRLA in groups with different types of TCGA‐RCC clinicopathologic data. The correlation between FCRLA expression and clinicopathological data was analyzed. All plots were analyzed through the R package “ggplot2” (v3.3.3).^24^

### 2.3 Kyoto Encyclopedia of Genes and Genomes (KEGG), Gene Ontology (GO) enrichment, and protein–protein interaction (PPI) network analysis

The co‐expression genes of FCRLA in the TCGA‐RCC database were obtained through LinkedOmics (http://www.linkedomics.org/).^25^ Based on the co‐expressed genes, the top 200 related genes were uploaded to the Metascape platform (http://metascape.org)^26^ for GO and KEGG enrichment analyses. With the gene numbers set from 5 to 5000; *p‐*value <0.05 and a false discovery rate <0.1 were considered statistically significant. The enrichment items were visualized by the R package “ggplots2.” Moreover, the STRING database (https://cn.string‐db.org/)^27^ was used for PPI network analysis, setting the interaction score to 0.4. The tool Cytoscape (version 3.8.2) was used for network depiction.

### 2.4 Cell culture

Two RCC cell lines 786‐O and ACHN were purchased from the Procell Life Science Technology Company (China) and cultured with complete Dulbecco's modified Eagle's medium (DMEM, Procell Life Science & Technology, China) with 10% fetal bovine serum and 1% penicillin–streptomycin in an incubator containing 5% CO_2_ at 37°C. Cells were passaged when 80%–90% confluence was reached. To construct FCRLA‐knockdown (KD) and negative control (NC) cells, lentivirus supernatant was used to infect 786‐O and ACHN cells, followed by selection in puromycin.

### 2.5 RNA extraction and qRT‐PCR

Using Trizol reagent (Shanghai Pufei Biotech, Shanghai, China), total RNA was isolated from the cells. cDNA was made using a PrimeScript RT reagent kit (Takara, RR047A). A SYBR Premix Ex Taq kit (Takara, RR820A) was used for qRT‐PCR, which was performed on an ABI 7500 real‐time PCR system (Applied Biosystems, Foster City, CA, USA). The profile of qRT‐PCR was as follows: 95°C for 30 s, 40 cycles of 95°C for 5 s and 60°C for 30 s, and a final extension of 72°C for 10 min. GAPDH was used as an endogenous control for qRT‐PCR. The 2 delta C_t_ method was used for the analysis of the PCR results. Primer sequences were as follows: GAPDH forward, TGACTTCAACAGCGACACCCA; reverse, CACCCTGTTGCTGTAGCCAAA; FCRLA forward, CGGAGGATGACTTGACTGATG; reverse, TGTACCACGGTGATGGAGAA.

### 2.6 Western blotting

Cells were harvested and then lysed, and extracts were subjected to sodium dodecyl sulfate–polyacrylamide gel electrophoresis and transferred to polyvinylidene difluoride membranes (Thermo Fisher Scientific, USA). After blocking with skimmed milk for 1 h at room temperature, membranes were incubated with primary antibodies (Table 1) overnight at 4°C. Subsequently, membranes were washed and incubated with goat anti‐rabbit or anti‐mouse secondary antibodies (Cat. #7074, #7076; CST) at room temperature for 1 hour. Protein bands were visualized using an enhanced chemiluminescence system (Thermo Fisher, 32,106).

**TABLE 1:** Primary antibodies used in this study.

Primary antibody	Source species	Company	Product No.	Predicted band size	Dilution
ERK1/2	Mouse	CST	#9107	42, 44 kDa	1:1000
MMP2	Rabbit	PROTEIN TECH	10,373‐2‐AP	72 kDa	1:500
p‐ERK1/2	Rabbit	CST	#4376	42, 44 kDa	1:1000
GAPDH	Mouse	SANTA CRUZ	sc‐32,233	36 kDa	1:2000
FCRLA	Mouse	SANTA CRUZ	sc‐53,583	40 kDa	1:1000

### 2.7 Cell proliferation assay

Three thousand cells were seeded in each well of a 96‐well plate, with four replicates for each group. Viability was assessed daily over 5 days, using an MTT kit (Glenview, USA) according to the manufacturer's instructions. MTT solution was added (10 μL/well), and cells were incubated for 1 h. OD was measured with a microplate reader (Tecan Infinite, Switzerland) at 490 nm.

### 2.8 Migration and invasion assay

To perform the migration assay, 24‐well transwell plates (Corning, NY, USA) were used. Briefly, the upper chamber of a transwell was precoated with 100 μg of Matrigel, then 1 × 10^4^ cells in 100 μL of FBS‐free DMEM were seeded into the upper chamber, while 500 μL of complete DMEM was added to the lower chamber as a chemoattractant. After incubating the cells for 20 h, nonmigratory cells on the upper chamber were removed using a cotton swab. The remaining cells were fixed and stained with crystal violet for 20 min, followed by capturing images of three random fields under a microscope (200× magnification). The invasion assay followed the same procedure, except Matrigel was not used to coat the upper chamber.

### 2.9 Apoptosis assay

Apoptotic cells in each group were measured using an apoptosis detection kit (Thermo Fisher Scientific, USA). Following three rounds of phosphate‐buffered saline washing, the cells were incubated with dyes for 15 min (avoiding light). A C6 PLUS flow cytometer (BD, USA) was used to count the number of apoptotic cells.

### 2.10 Immune infiltration analysis

The “GSEA” function in R package “GSVA”^28^ was applied to investigate the relationship between FCRLA and 24 types of common immune‐infiltrating cells, and the results were evaluated by the Spearman test. Subsequently, the results were validated by the TIMER algorithm. The relationship between immune gene markers and FCRLA was also assessed using the GEPIA (http://gepia.cancer‐pku.cn/index.html) and TIMER (Version 2.0) databases.

### 2.11 Tumor purity and immune therapy response analysis

Stromal scores, immune scores, and ESTIMATE scores in the high and low FCRLA expression groups were determined by the Estimation of Stromal and Immune cells in Malignant Tumor Tissues using the Expression data (ESTIMATE) algorithm.^29^ The immune therapy response index was assessed according to the dysfunction score, exclusion score, and tumor immune dysfunction and exclusion (TIDE) score through the TIDE algorithm.^30^

### 2.12 Statistical analysis

Each experiment was performed three times, and the SPSS statistical software package (SPSS22.0, USA) and GraphPad Prism software (GraphPad 6.0, USA) were used to plot the curves. A *p*‐value < 0.05 indicated statistical significance. Data were displayed to be the mean ± standard deviation from three separate assays.

## 3 RESULTS

### 3.1 Highly expressed FCRLA was correlated with T stage, N stage and poor prognosis in RCC patients

**FIGURE 1:**
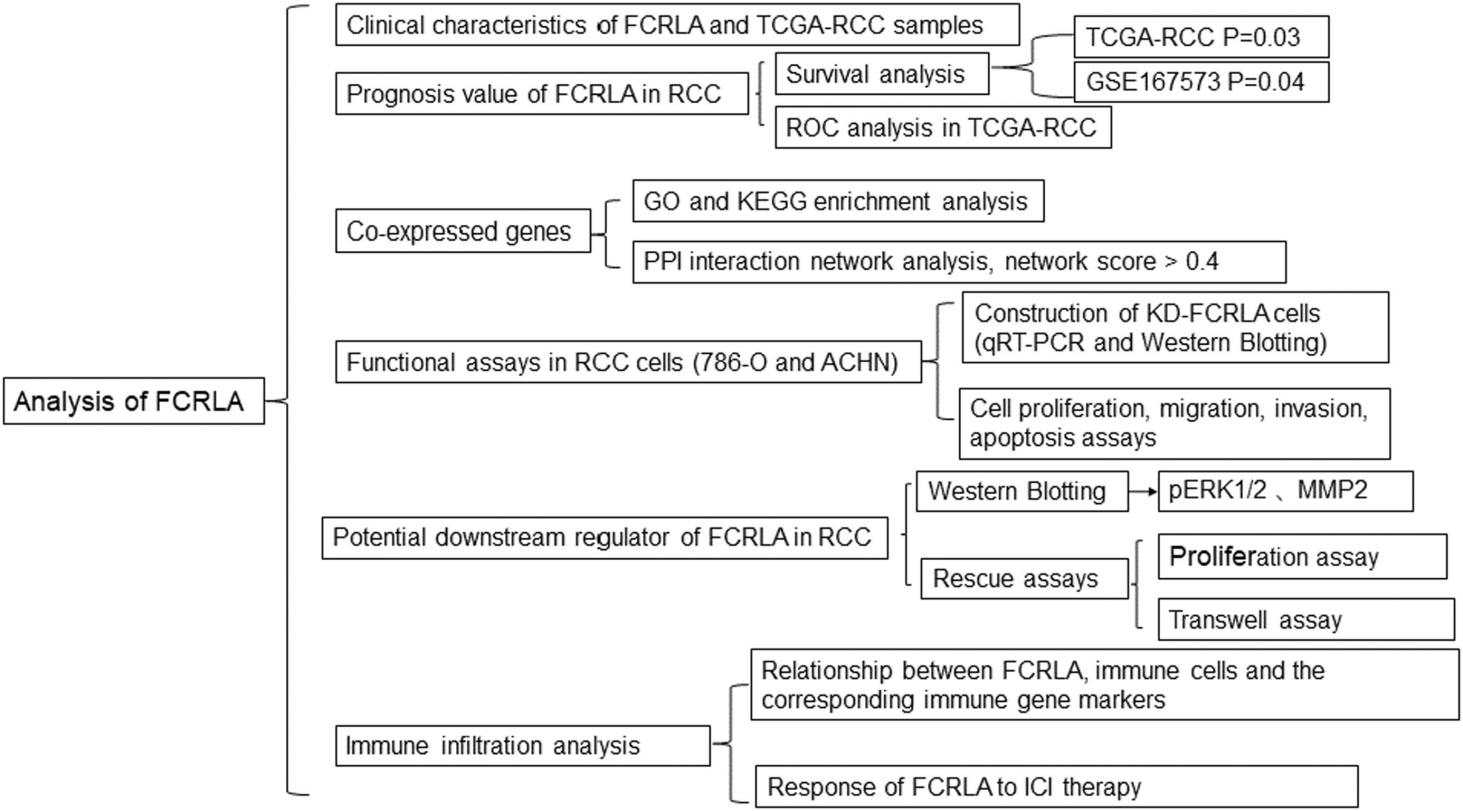
Flow chart of methodologies used in this study.

**FIGURE 2:**
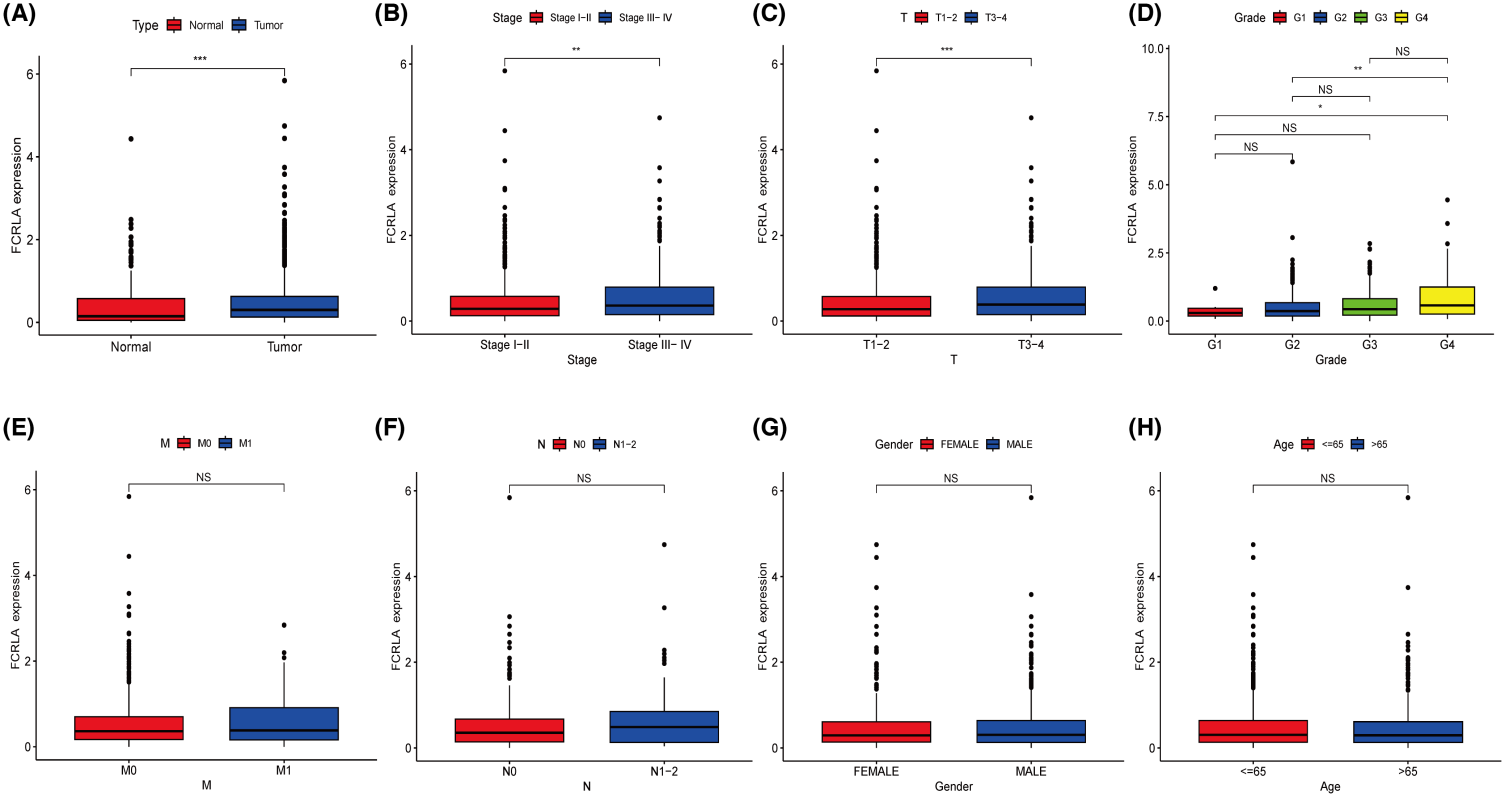
Bioinformatics analysis of FCRLA in renal cell carcinoma (RCC) patients. (A) FCRLA mRNA expression between tumor tissues and adjacent normal tissues in TCGA‐RCC. (B) Difference in *FCRLA* mRNA expression between advanced stage (stage III and IV) patients and early stage (stage I and II) patients. (C) Difference in *FCRLA* mRNA expression between T1–2 stages and T3–4 stages. (D) Difference in *FCRLA* mRNA expression between pathological grades. (E‐H) Difference in *FCRLA* mRNA expression between M stage, N stage, gender and age. *p < 0.05, **p < 0.01, ***p < 0.001 and NS: Not significant.

The flow chart of the methodologies used in this study is shown in Figure 1. All clinicopathological data of the 886 patients recorded in TCGA‐RCC, including their tumor–lymph node–metastasis (TNM) stage, pathological grade, gender, clinical stage, and age, are listed in Table 2. FLRLA gene expression was higher in RCC tumor comparing to normal samples (Figure 2A). The results demonstrated that the FCRLA expression level in advanced clinical stage (stage III and IV) patients was higher than that in early stage patients (stage I and II) (Figure 2B). RCC patients in the T3 and T4 stages had greater FCRLA expression compared to those in the T1 and T2 stage (Figure 2C). Similarly, FCRLA in RCC patients with pathological grade 4 was higher than that in those with grade 2 and grade 1 (Figure 2D). FCRLA levels did not show any statistically significant differences when compared M stage, N stage, gender, and age (Figure 2E–H).

**TABLE 2:** Clinical and pathological data of the patients in the TCGA‐RCC cohort.

Characteristics	Low expression of FCRLA	High expression of FCRLA	*p*‐Value
*n*	443	443	
Age, median (IQR)	61 (52, 70)	60 (51, 69)	0.179
Gender, *n* (%)			0.775
Female	147 (33.21)	143 (32.18)	
Male	296 (66.79)	300 (67.82)	
Pathological Grade, *n* (%)			<0.001
G1	7 (1.60)	7 (1.60)	
G2	95 (21.42)	134 (30.21)	
G3	77 (17.47)	129 (29.13)	
G4	22 (4.82)	54 (12.17)	
Unknown	242 (54.69)	119 (26.89)	
Clinical Stage, *n* (%)			0.122
Stage I	240 (54.21)	219 (49.32)	
Stage II	52 (11.73)	50 (11.28)	
Stage III	79 (17.87)	109 (24.60)	
Stage IV	52 (11.76)	52 (11.73)	
Unknown	20 (4.43)	13 (3.07)	
T stage, *n* (%)			0.045
T1	258 (58.24)	228 (51.52)	
T2	67 (15.12)	59 (13.32)	
T3	110 (24.83)	147 (33.18)	
T4	8 (1.81)	7 (1.58)	
Unknown	0 (0)	2 (0.4)	
M stage, *n* (%)			< 0.001
M0	238 (53.72)	311 (70.20)	
M1	40 (9.02)	50 (11.28)	
Unknown	165 (37.26)	82 (18.42)	
N stage, *n* (%)			0.025
N0	148 (33.40)	182 (41.08)	
N1	17 (3.84)	26 (5.87)	
N2	4 (0.90)	2 (0.45)	
Unknown	274 (61.86)	233 (52.60)	

**FIGURE 3:**
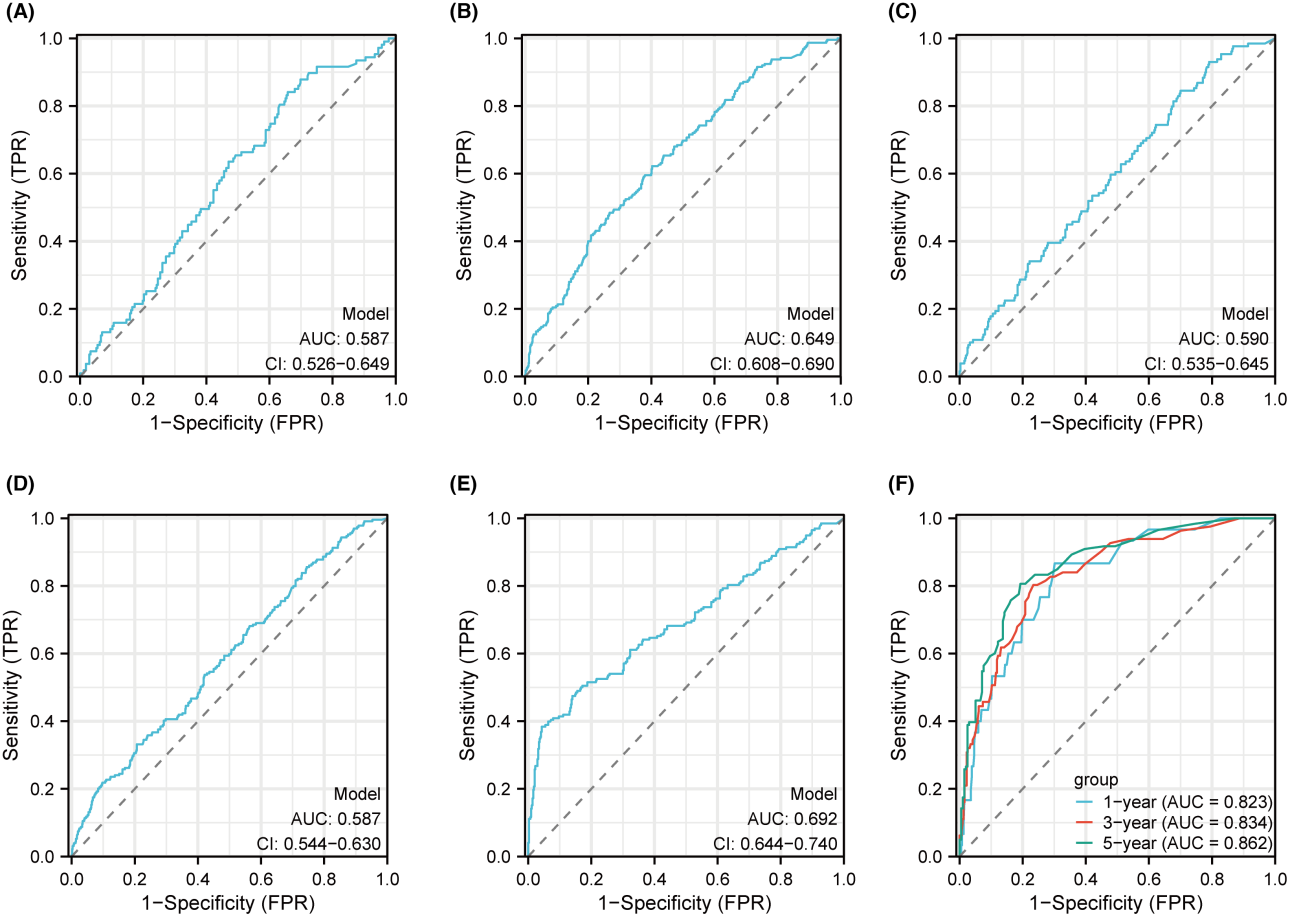
Receiver operating characteristic (ROC) curves of *FCRLA* for different clinical parameters of renal cell carcinoma patients. ROC curves indicate the correlation of *FCRLA* expression with N events (A), clinical stage (B), pathological grade (C), T stage (D), M stage (E), and 1‐, 3 and 5‐year survival rates (F).

**FIGURE 4:**
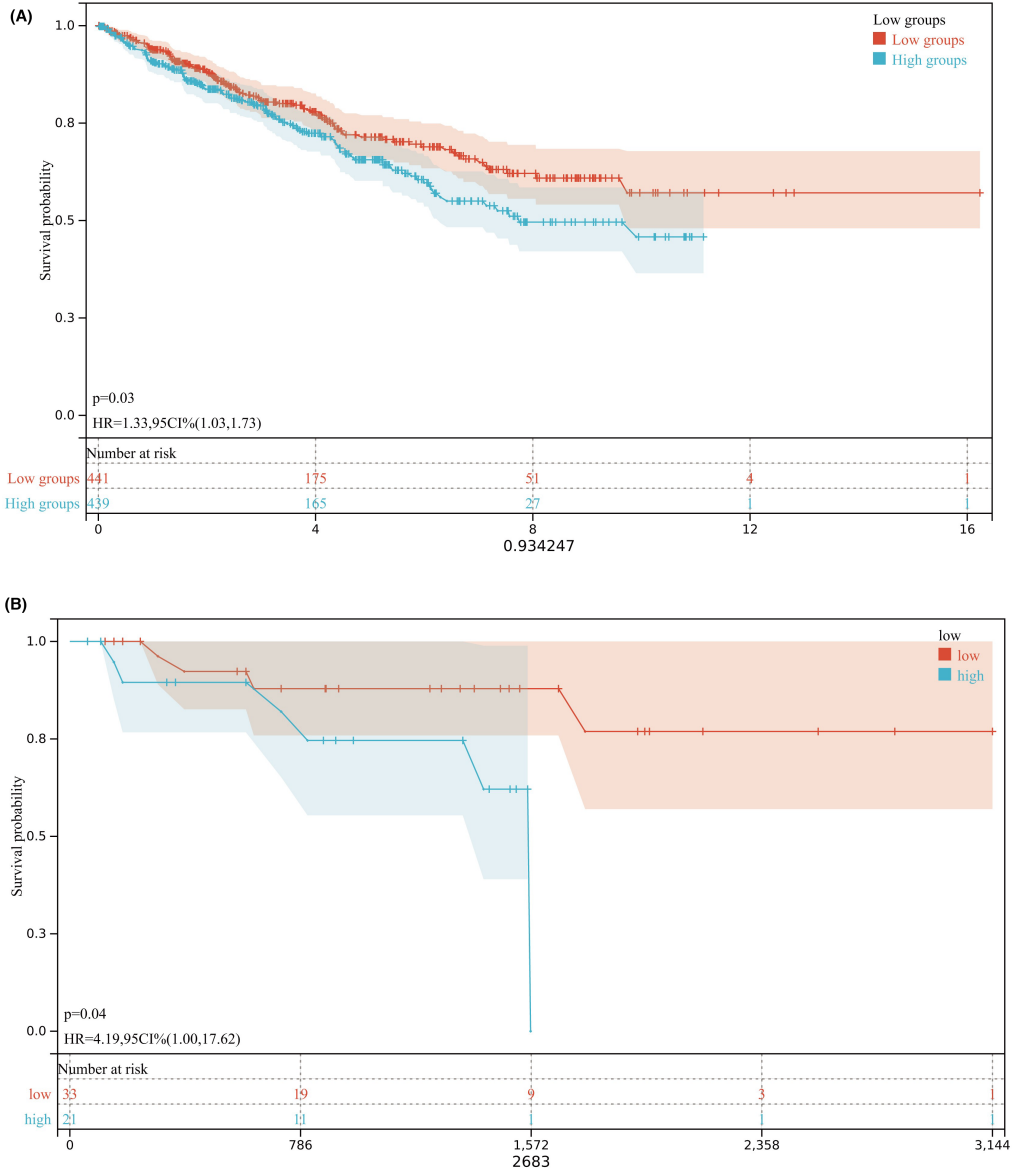
Kaplan–Meier curves depict higher expression of FCRLA leads to worse prognosis in RCC. From TCGA (A) and GSE 167573 (B).

ROC curves demonstrated the predictive ability of FCRLA in RCC cases. The predictive ability of FCRLA expression in the N stage (area under curve, AUC = 0.587), clinical stage (AUC = 0.649), pathological grade (AUC = 0.590) and T stage (AUC = 0.587) was not satisfactory (Figure 3A–D). The calculated AUC indicating FCRLA had a high level of discriminatory accuracy in predicting the M stages (AUC = 0.692, Figure 3E), 1‐, 3‐, and 5‐year survival rates (AUC = 0.823, 0.834, and 0.862, respectively, Figure 3F). OS curves for TCGA‐RCC patients showed patients with lower expression of FCRLA had significantly longer OS (*p* = 0.03, HR = 1.33, 95% CI = 1.03–1.73; Figure 4A) than patients with higher FCRLA expression. Similar results were observed in GSE167573 (*p* = 0.04, HR = 1.33, 95% CI = 1.00–17.62; Figure 4B).

### 3.2 Enrichment analyses of FCRLA and its co‐expressed genes

**FIGURE 5:**
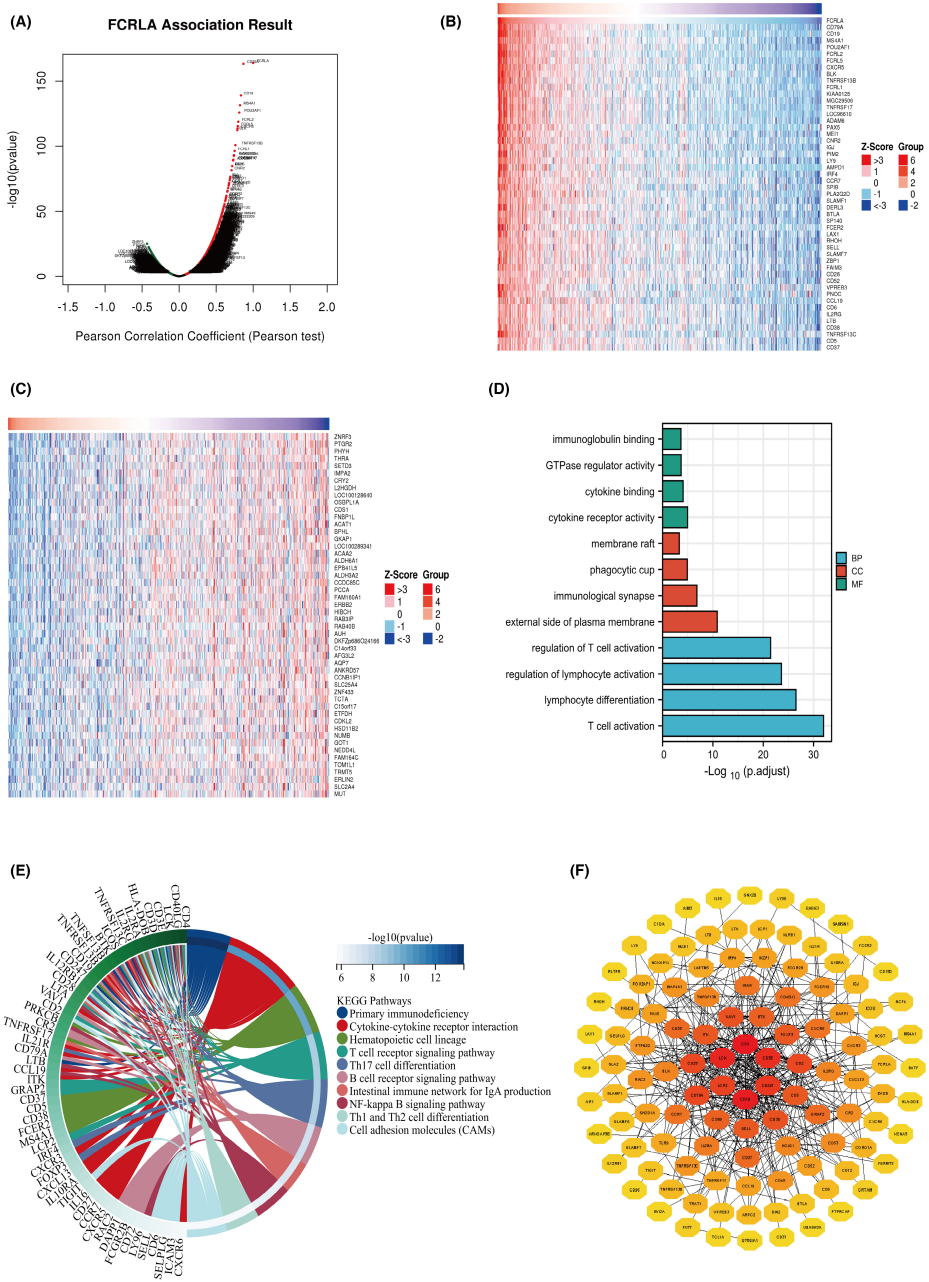
Analysis of *FCLRA* and its co‐expressed genes. (A) Positively and negatively correlated co‐expressed genes of FCRLA are shown in the volcano map. (B, C) Heat maps of the top 50 genes positively or negatively associated with FCRLA expression. (D) Gene Ontology (GO) enrichment analysis of FCRLA in biological processes, cellular components, and molecular functions. (E) Kyoto Encyclopedia of Genes and Genomes (KEGG) pathway analysis of FCRLA. (F) Protein–protein interaction network of FCRLA with the top 200 co‐expressed genes.

To examine FCRLA's potential mechanisms in RCC in more detail, the online‐tool LinkedOmics (http://www.linkedomics.org) was used to identify FCRLA co‐expressed genes. The analysis showed 7027 positively correlated genes and 3707 negatively correlated genes (Figure 5A). Heat maps listed the 50 genes with the highest positive (Figure 5B) and negative (Figure 5C) correlations.

Enrichment analyses were performed based on FCRLA and its top 200 negatively and positively co‐expressed genes. GO analysis indicated that FCRLA as well as its co‐expressed genes were mainly enriched in the following pathways: immunoglobulin binding, GTPase regulator activity, cytokine binding, cytokine receptor activity, membrane raft, phagocytic cup, immunological synapse, external side of the plasma membrane, regulation of T cell activation, regulation of lymphocyte activation, lymphocyte differentiation, and T cell activation (Figure 5D). KEGG enrichment items indicated that FCRLA and its co‐expressed genes were mainly enriched in the following pathways: primary immunodeficiency, cytokine‐cytokine receptor interaction, hematopoietic cell lineage, T cell receptor signaling pathway, T helper (Th) 17 cell differentiation, B cell receptor signaling pathway, the intestinal immune network for IgA production, NF‐kappa B signaling pathway, Th1 and Th2 cell differentiation, and cell adhesion molecules (Figure 5E). The PPI network indicated there were 126 nodes and 425 edges correlated with FCRLA (Figure 5F).

### 3.3 FCRLA knockdown inhibits proliferation, migration, and invasion of RCC cells, inducing tumor cell apoptosis

**FIGURE 6:**
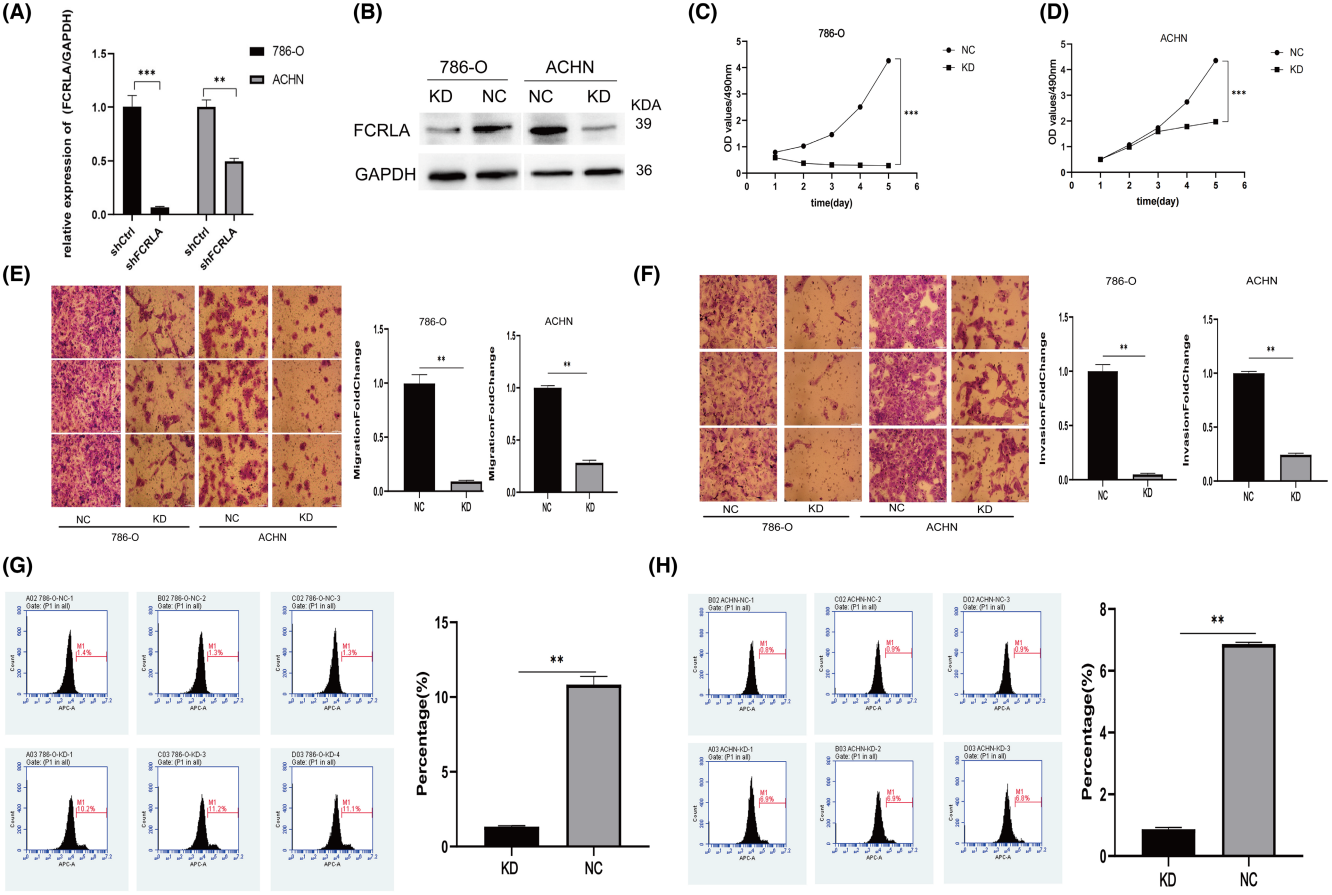
*FCRLA* expression in different renal cell carcinoma (RCC) cell lines and its influence on different RCC cell lines as measured by functional assays. (A, B) The mRNA and protein expression levels of *FCRLA* in 786‐O/knockdown (KD), 786‐O/normal control (NC) cells, ACHN/KD, and ACHN/NC cells. (C, D) Cell viability was reduced in FCRLA‐KD RCC cell lines compared to that in controls. (E, F) Inhibition of cell migration and invasion after *FCRLA* KD in 786‐O and ACHN cells, as determined by a transwell assays. (G, H) The number of apoptotic cells increases after *FCRLA* KD in 786‐O and ACHN cells. *p < 0.05, **p < 0.01, and ***p < 0.001.

Both qRT‐PCR and western blotting confirmed a significant decrease of FCRLA expression in both KD groups of cells (Figure 6A,B). The results also verified that the viability of KD cells was significantly decreased in both 786‐O and ACHN cells (Figure 6C,D). Transwell assays demonstrated decreased migration and invasion capabilities of KD cells compared to NC cells (*p* < 0.05; Figure 6E,F). The percentage of apoptotic cells in KD cells was greater than that in NC cells, suggesting that FCRLA may suppress apoptosis in RCC cells (Figure 6G,H). These findings collectively show that FCRLA knockdown could inhibit the development of RCC.

### 3.4 FCRLA induces the proliferation, migration, and invasion of RCC cells by regulating p‐ERK1/2/MMP2 expression

**FIGURE 7:**
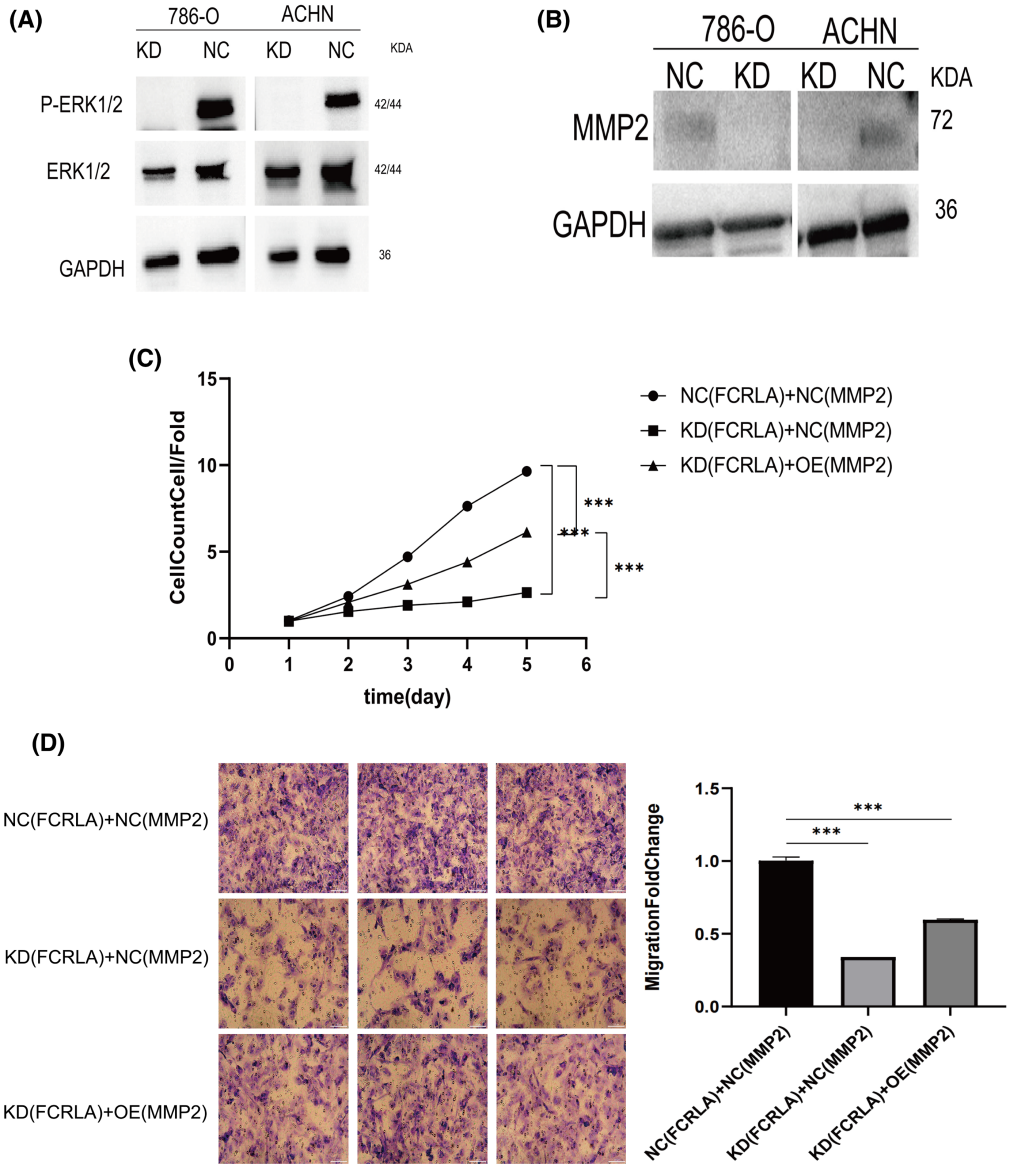
FCRLA affects the expression of MMP2 by regulating pERK/2. (A) P‐ERK1/2 and ERK1/2 protein levels in 786‐O/KD, 786‐O/NC, ACHN/KD, and ACHN/NC cells were quantitatively analyzed by western blotting. (B) MMP2 protein levels in four groups of cells were quantitatively analyzed by western blotting. (C) Overexpression of MMP2 partially reverses the inhibition of proliferation caused by FCRLA in 786‐O/KD cells, as measured by MTT assay. (D) Overexpression of MMP2 can partially reverse the inhibition of the migration ability by FCRLA in 786‐O/KD cells as measured by a trans well assay. ***P<0.001.

In previous studies, we identified ERK1/2 as a potential upstream regulator inducing MMP2,^31^ which plays a crucial role in regulating the migration and invasion of RCC.^32^ In our current study, knockdown of FCRLA did not impact the expression level of ERK1/2 but significantly reduced the phosphorylation level of ERK1/2 (Figure 7A). However, the mechanism between MMP2 and FCRLA remains unclear. MMP2 expression is down‐regulated in the 786‐O/KD and ACHN/KD groups (Figure 7B). For rescue experiments, we focused on 786‐O cells. Both 786‐O/KD and 786‐O/NC cells were transfected with either lentiviral MMP2 or the corresponding NC, and proliferation and invasion of these transfected cells were examined by MTT or transwell assay. Based on the following three groups: FCRLA/NC + MMP2/NC, FCRLA/KD + MMP2/NC, and FCRLA/KD + MMP2/overexpressed (OE) (Figure 7C), the results demonstrated that overexpressing MMP2 in FCRLA‐KD RCC cells could partially reverse the inhibition of cell proliferation. Similarly, transwell assays indicated that MMP2 overexpression attenuated the reduced migration ability of 786‐O/FCRLA‐KD cells compared to the other cell lines (Figure 7D). In summary, our findings suggest that FCRLA promotes malignant behaviors in RCC in an MMP2‐dependent manner.

### 3.5 FCRLA correlates with immune infiltration and the response to immune checkpoint inhibitor therapy in RCC

**FIGURE 8:**
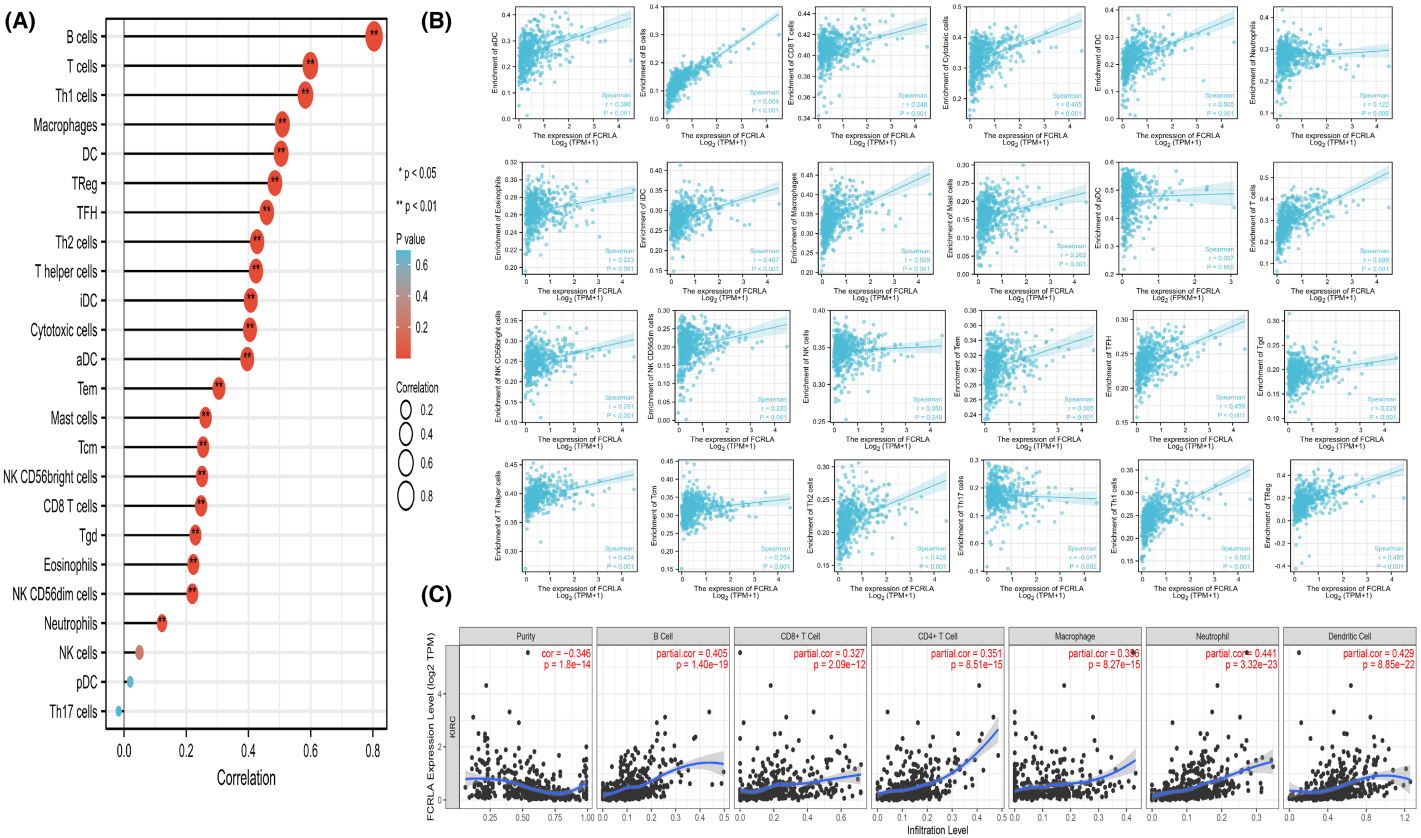
Single‐sample gene set enrichment analysis. (A) The association of FCRLA with 24 types of common immune cells. (B) Scatter plots showing the association of FCRLA expression with 24 immune‐infiltrating cell types. (C) The association of FCRLA with tumor purity and six types of immune‐infiltrating cells was analyzed by the TIMER algorithm. *p < 0.05, **p < 0.01, and ***p < 0.001.

**FIGURE 9:**
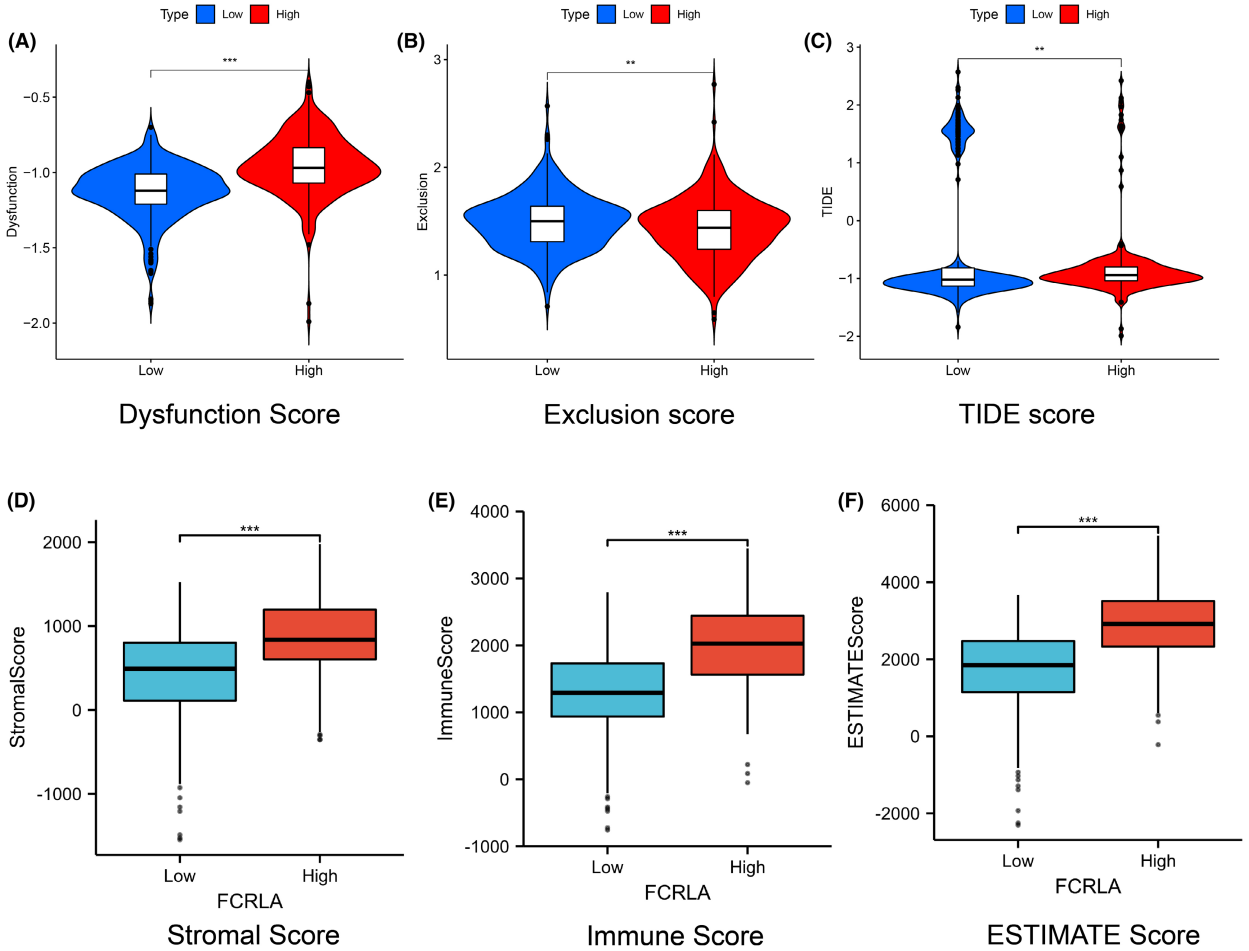
Analysis results of the tumor immune dysfunction and exclusion (TIDE) and Estimation of Stromal and Immune cells in Malignant Tumor tissues using Expression data (ESTIMATE) algorithms. The dysfunction score (A), exclusion score (B), and TIDE score (C) were assessed using the TIDE algorithm. The stromal score (D), immune score (E), and ESTIMATE score (F) were evaluated by the ESTIMATE algorithm. *p < 0.05, **p < 0.01, and ***p < 0.001.

We further investigated the impact of FCRLA on immune infiltration in RCC, examining its relationship with 24 types of immune cells as depicted in Figure 8A. As depicted in Figure 8B, 21 types of immune cells were positively associated with FCRLA expression, including anchorage‐dependent cells (*r* = 0.369, *p* = 2.24 × 10^−13^), B cells (*r* = 0.804, *p* = 3.5 × 10^−9^), CD8+ T cells (*r* = 0.248, *p* = 2.44 × 10^−9^), cytotoxic cells (*r* = 0.405, *p* = 3.07 × 10^−26^), dendritic cells (*r* = 0.505, *p* = 3.67 × 10^−31^), eosinophils (*r* = 0.223, *p* = 6.91 × 10^−5^), immature dendritic cells (*r* = 0.407, *p* = 2.03 × 10^−20^), macrophages (*r* = 0.509, *p* = 5.28 × 10^−33^), mast cells (*r* = 0.262, *p* = 4.5 × 10^−7^), neutrophils (*r* = 0.122, *p* = 0.005), natural killer/CD56 bright cells (*r* = 0.251, *p* = 3.68 × 10^−11^), natural killer/CD56 dim cells (*r* = 0.22, *p* = 1.03 × 10^−6^), T cells (*r* = 0.599, *p* = 8.48 × 10^−56^), T helper cells (*r* = 0.424, *p* = 1.15 × 10^−20^), central memory T cells (*r* = 0.254, *p* = 2.61 × 10^−7^), effective memory T cells (*r* = 0.305, *p* = 1.87 × 10^−11^), follicular helper T cells (*r* = 0.459, *p* = 7.11 × 10^−27^), gamma delta T cells (*r* = 0.229, *p* = 1.91 × 10^−5^), Th1 cells (*r* = 0.58, *p* = 1.01 × 10^−47^), Th2 cells (*r* = 0.428, *p* = 1.01 × 10^−22^), and regulator T cells (*r* = 0.485, *p* = 8.53 × 10^−37^). In addition, the associations of FCLRA with various gene markers of immune cells are listed in Table 3, revealing the correlation of FCRLA with the most common immune markers, such as CD8A, CD19, and CCR3. Figure 8C shows similar correlations of FCRLA expression with tumor purity (*r* = −0.346, *p* = 1.8 × 10^−14^), B cells (*r* = 0.405, *p* = 1.4 × 10^−19^), CD8+ T cells (*r* = 0.327, *p* = 2.09 × 10^−12^), CD4+ T cells (*r* = 0.351, *p* = 8.5 × 10^−15^), macrophages (*r* = 0.356, *p* = 8.27 × 10^−15^), neutrophils (*r* = 0.441, *p* = 3.32 × 10^−23^), and dendritic cells (*r* = 0.429, *p* = 8.85 × 10^−22^). It has been reported that a patient's response to immunotherapy can be inferred by the TIDE score.^27^ In our study, the groups with higher FCRLA expression had a higher degree of dysfunction and exclusion, as well as higher TIDE score (Figure 9A–C). The ESTIMATE algorithm was employed to evaluate the association of FCRLA with tumor stromal cells and tumor immune cells, which may provide a new reference for immune therapy due to the enrichment of FCRLA in tumor stromal cells and immune cells (Figure 9D–F). In short, higher FCRLA expression in RCC patients may respond better to immune checkpoint inhibitor therapy.

**TABLE 3:** Correlation of FCRLA expression with markers of immune cells using analysis of the TIMER and GEPIA databases.

Cell type	Gene marker	None		Purity		Tumor		Normal	
		Cor	*p*	Cor	*p*	Cor	*p*	Cor	*p*
B cell	CD19	0.765	***	0.741	***	0.93	***	0.86	***
	CD20 (KRT20)	0.142	**	0.118	**	−0.012	0.79	0.17	0.15
	CD38	0.566	***	0.535	***	0.23	***	0.62	***
CD8^+^ T cell	CD8A	0.456	***	0.412	***	0.066	0.13	0.71	***
	CD8B	0.424	***	0.379	***	0.067	0.13	0.52	***
Tfh	BCL6	0.051	0.24	0.077	0.0978	0.036	0.41	−0.094	0.43
	ICOS	0.51	***	0.493	***	0.12	**	0.61	***
	CXCR5	0.713	***	0.699	***	0.17	***	0.68	***
Th1	T‐bet (TBX21)	0.294	***	0.241	***	0.23	***	0.36	**
	STAT4	0.418	***	0.38	***	0.23	***	0.59	***
	IL12RB2	0.252	***	0.23	***	−0.02	0.64	0.35	**
	WSX1 (IL27RA)	0.138	**	0.0062	0.185	0.054	0.22	0.18	0.13
	STAT1	0.387	***	0.344	***	0.075	0.088	0.16	0.18
	IFN‐γ (IFNG)	0.403	***	0.347	***	0.056	0.2	0.23	0.051
	TNF‐α (TNF)	0.274	***	0.245	***	0.057	0.19	0.26	*
Th2	GATA3	0.278	***	0.287	***	−0.009	0.84	−0.29	*
	CCR3	0.279	***	0.226	***	0.032	0.47	0.48	***
	STAT6	−0.051	0.236	−0.046	0.324	0.13	**	0.19	0.12
	STAT5A	0.436	***	0.396	***	0.13	**	0.26	*
Th9	TGFBR2	0.021	0.627	−0.021	0.648	−0.021	0.64	0.57	***
	IRF4	0.651	***	0.621	***	0.44	***	0.71	***
	PU.1 (SPI1)	0.499	***	0.447	***	0.14	**	0.58	***
Th17	STAT3	0.137	**	0.1	*	0.039	0.38	0.16	0.18
	IL21R	0.535	***	0.49	***	0.12	**	0.65	***
	IL23R	0.331	***	0.29	***	0.037	0.39	0.22	0.064
	IL17A	0.194	***	0.184	***	0.19	***	0.22	0.058
Th22	CCR10	0.094	*	0.022	0.638	0.018	0.68	0.2	0.086
	AHR	0.153	***	0.117	*	−0.006	0.89	0.29	*
Treg	FOXP3	0.533	***	0.502	***	0.15	***	0.26	*
	CD25 (IL2RA)	0.498	***	0.471	***	0.14	**	0.69	***
	CCR8	0.489	***	0.457	***	0.11	*	0.54	***
T cell exhaustion	PD‐1 (PDCD1)	0.444	***	0.41	***	0.092	*	0.64	***
	CTLA4	0.462	***	0.443	***	0.34	***	0.47	***
	LAG3	0.405	***	0.368	***	0.083	0.059	−0.084	0.48
	TIM‐3 (HAVCR2)	0.175	***	0.137	**	−0.016	0.72	0.47	***
Macrophage	CD68	0.33	***	0.315	***	0.041	0.35	0.29	*
	CD11b (ITGAM)	0.389	***	0.348	***	0.048	0.28	0.48	***
M1	INOS (NOS2)	0.052	0.234	−0.026	0.574	0.23	0.05	−0.0016	0.97
	IRF5	0.186	***	0.167	***	0.12	**	−0.27	*
	COX2 (PTGS2)	0.241	***	0.21	***	0.014	0.76	−0.023	0.85
M2	CD163	0.408	***	0.383	***	0.13	**	0.53	***
	ARG1	−0.011	0.801	0.017	0.708	0.0085	0.85	0.17	0.15
	MRC1	0.202	***	0.152	**	0.048	0.27	0.64	***
	MS4A4A	0.461	***	0.425	***	0.14	**	0.58	***
TAM	CCL2	0.093	*	0.049	0.29	−0.042	0.34	0.36	**
	CD80	0.458	***	0.458	***	0.13	**	0.23	*
	CD86	0.506	***	0.484	***	0.087	*	0.55	***
	CCR5	0.489	***	0.452	***	0.086	*	0.54	***
Monocyte	CD14	0.448	***	0.39	***	0.063	0.15	0.51	***
	CD16 (FCGR3B)	0.157	***	0.158	***	0.021	0.64	0.15	0.2
	CD115 (CSF1R)	0.433	***	0.384	***	0.097	*	0.52	***
Neutrophil	CD66b (CEACAM8)	0.019	0.657	0.032	0.488	0.28	***	−0.032	0.79
	CD15 (FUT4)	0.253	***	0.218	***	0.083	0.058	0.59	***
	CD11b (ITGAM)	0.389	***	0.348	***	0.048	0.28	0.48	***
Natural killer cell	XCL1	0.468	***	0.432	***	0.14	***	0.47	***
	CD7	0.473	***	0.42	***	0.011	0.8	0.011	0.8
	KIR3DL1	0.091	*	0.072	0.123	−0.04	0.37	0.35	**
Dendritic cell	CD1C (BDCA‐1)	0.437	***	0.376	***	0.18	***	0.84	***
	CD141 (THBD)	0.203	***	0.103	*	0.07	0.11	0.42	***
	CD11c (ITGAX)	0.364	***	0.355	***	0.19	***	0.46	***

*Note*: *p < 0.05; **p < 0.01; ***p < 0.001.

Abbreviations: Cor, R‐value of Spearman's correlation; None, correlation without adjustment; Purity, correlation adjusted by purity; TAM, Tumor‐associated macrophage; Tfh, Follicular helper T cells; Th, T helper cells; Treg, Regulatory T cells.

## 4 DISCUSSION

We found that FCRLA expression is highly expressed in advanced renal carcinoma compared to other stages of RCC. Aberrant FCRLA expression may predict poor OS for RCC patients. FCRLA promotes the proliferation, migration, and invasion of RCC cells and inhibits tumor cell apoptosis. Furthermore, MMP2 could partially reverse the inhibition of proliferation and migration caused by FCRLA KD in RCC cells. FCRLA may regulate MMP2 by affecting pERK1/2. Our study conclusively demonstrates that FCRLA plays a crucial role in enhancing the proliferation, invasion, and migration of RCC cells as evidenced by a series of rigorous experiments. These findings firmly establish FCRLA as a key regulator in the progression of renal cell carcinoma (RCC). Specifically, the observed inhibition of cell proliferation upon FCRLA knockdown underscores its significant role as a pro‐proliferative factor in RCC. Furthermore, our results indicate that modulation of FCRLA levels enhances the invasion and migration capabilities of RCC cells, suggesting FCRLA's involvement in promoting metastatic traits. This study represents the first exploration of FCRLA's biological role in malignant tumors, expanding beyond previous research focused on its immunological implications. Insights gained from our experiments suggest that FCRLA may influence critical signaling pathways involved in RCC pathogenesis, potentially offering new therapeutic targets or prognostic markers. Further investigation into the specific molecular mechanisms through which FCRLA mediates its effects on RCC progression is warranted to fully elucidate its role and therapeutic implications.

FCRLA is highly enriched not only in RCC cells but also in the immune and stromal cells of the RCC microenvironment. It is positively associated with immune infiltrating cells, suggesting its potential value as an immunotherapy target. However, the TIDE score indicates that high FCRLA expression may predict a higher likelihood of immune escape, which could lead to the failure of immune checkpoint inhibitor (ICB) treatment. FCRLA is preferentially expressed in B cells and participates in immune response‐related pathways.^33^, ^34^ It has been identified as a potential target antigen in immunotherapy for B‐cell lymphoma.^35^ Additionally, RCC tissues contain more B cells compared to surrounding normal renal tissues, indicating that B cell accumulation in RCC may be associated with tumor metastasis.^36^ Elevated levels of CD8+ T cells, which are associated with poor prognosis in RCC, are also positively related to FCRLA,^37^ are also positively related to FCRLA. In our study, enrichment analysis showed that FCRLA and its co‐expressed genes are involved in the regulation of T cells, the immunological synapse, and T and B cell signaling pathways. This implies that targeting FCRLA in RCC could potentially be approached through T and B cell regulation.^38^ The TIDE algorithm further suggested that FCRLA might influence RCC progression through immune dysfunction and immune exclusion mechanisms, which are critical factors in poor immunotherapy outcomes.^39^ Despite this, the immune score suggests a close relationship between FCRLA and immune infiltrating cells in the RCC microenvironment, indicating potential for the development of new immune checkpoint inhibitors targeting FCRLA. Research has shown that a higher stromal score is positively associated with worse survival prognosis in RCC.^40^ Our results similarly indicate that higher FCRLA expression may lead to increased stromal cell infiltration in the tumor microenvironment of RCC patients, contributing to poor prognosis. The ESTIMATE score, which combines the immune and stromal scores, suggests that RCC patients with higher FCRLA expression have worse prognosis but a higher abundance of immune infiltrating cells, which might make them more responsive to immunotherapy. Thus, developing immune checkpoint inhibitors targeting FCRLA is crucial. The amount of extracellular matrix is primarily regulated by MMPs and tissue inhibitors of metalloproteinase, with MMP2 shown to be involved in tumor development.^41^ As a downstream target of FCRLA, MMP2 may provide valuable insights into the regulation of the RCC tumor microenvironment.^42^ Additionally, ERK1/2 dephosphorylation could be considered a therapeutic target in RCC.^43^

Our study revealed that FCRLA plays an oncogenic role in RCC development through a mechanism involving MMP2, indicative that a FCRLA‐*p*ERK1/2‐MMP2 signaling pathway could be a potential target for RCC immunotherapy.

Nevertheless, our study has some limitations, including the absence of in vivo animal studies and validation experiments using clinical specimens. Furthermore, the specific role of FCRLA in RCC warrants further investigation.

## 5 CONCLUSION

Our study reveals that FCRLA is highly expressed in the advanced RCC stages (T3, T4 stages, clinical stages III and IV) and predicts an unfavorable prognosis. FCRLA might be an acceptable marker for predicting the M stage and 1‐, 3‐, and 5‐year survival rates of RCC patients. Aberrant FCRLA expression can promote malignant biological behaviors of RCC, with suppression of tumor cell apoptosis in an MMP2‐dependent manner. FCRLA may regulate the expression of MMP2 by affecting the phosphorylation level of ERK1/2. Furthermore, FCRLA expression is associated with immune infiltration and the response to immune checkpoint inhibitor therapy in RCC patients.

## AUTHOR CONTRIBUTIONS

**Jun‐peng Liu:** Writing – original draft (equal). **Yi‐fan Jiang:** Validation (equal); visualization (equal). **Jin‐wen Liu:** Writing – review and editing (equal). **Chong‐jiang Tian:** Data curation (equal); software (equal). **Yu‐zhao Lin:** Validation (equal); writing – review and editing (equal). **Yun‐zhi Yang:** Methodology (equal). **Ze‐ke Zhang:** Data curation (equal). **Yi‐liang Fang:** Formal analysis (equal). **Bin Huang:** Funding acquisition (equal). **Hao Lin:** Funding acquisition (equal).

## FUNDING INFORMATION

This work was supported by the Medical Scientific Research Foundation of Guangdong Province (Grant Number: B2022311) and the National Natural Science Foundation of China (Grant Number: 82072817).

## CONFLICT OF INTEREST STATEMENT

The authors declare that they have no competing interests.

## CONSENT FOR PUBLICATION

We have obtained consent to publish this paper from all participants of this research.

## Supporting information


Figure S1.

## Data Availability

The bioinformatics data used in this study are freely available from the TCGA database (https://portal.gdc.cancer.gov/projects/TCGA‐RCC) and the GEO database (https://www.ncbi.nlm.nih.gov/geo/query/acc.cgi?acc=GSE167573). The authors did not have special access privileges.
