# Supplementary figures and images for: Fc receptor‐like A promotes malignant behavior in renal cell carcinoma and correlates with tumor immune infiltration
Source: Cancer Med. 2024 Aug 6;13(15):e70072. doi: 10.1002/cam4.70072 (PMC11303447; doi:10.1002/cam4.70072)

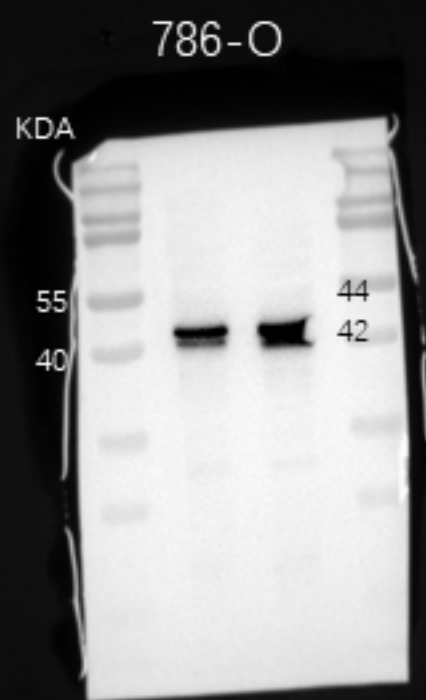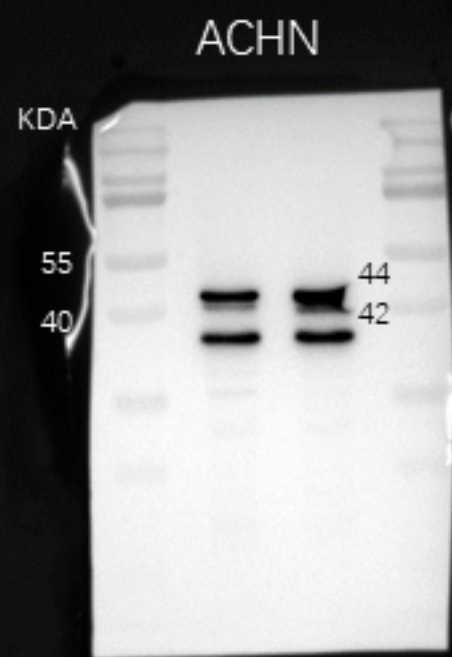

ERK1/2

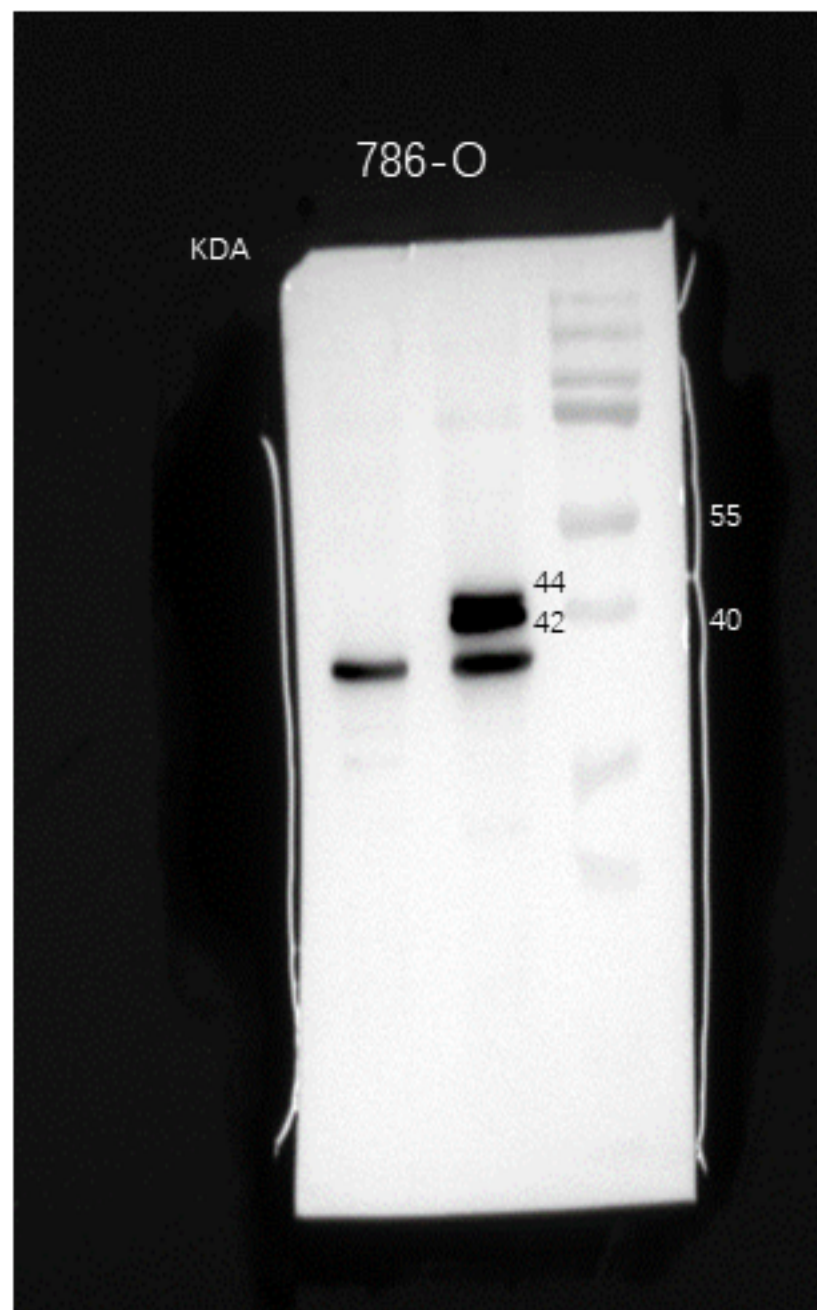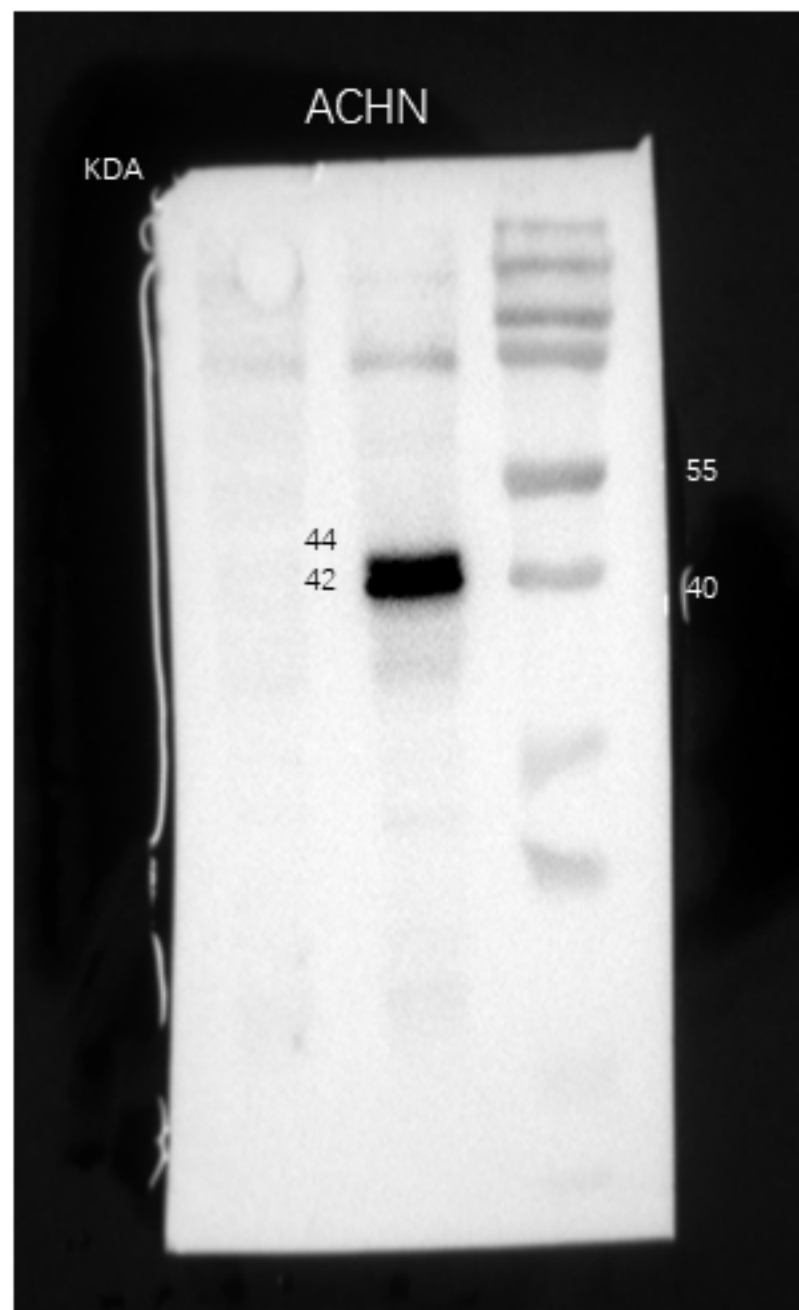

P-ERK1/2

786-O

KDA

70

55

40

36

ACHN

KDA

70

55

40

36

GAPDH

KDA

70

55

37

39

FCRLA

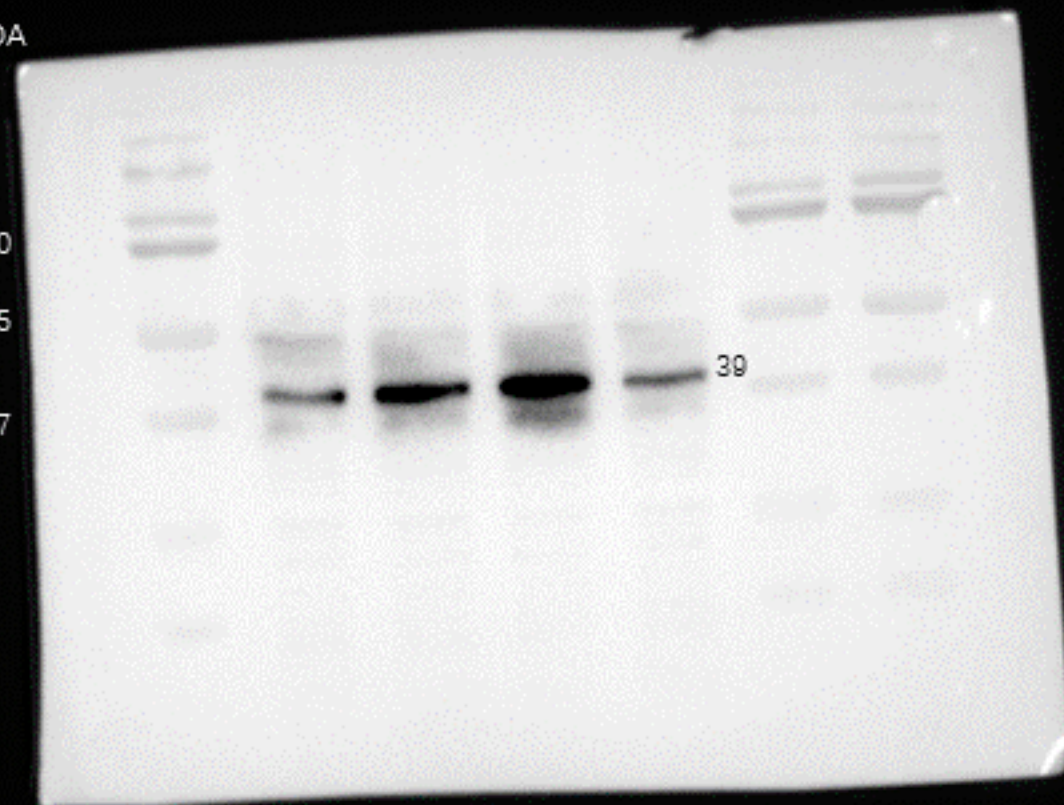

KDA

70

55

40

36

GAPDH

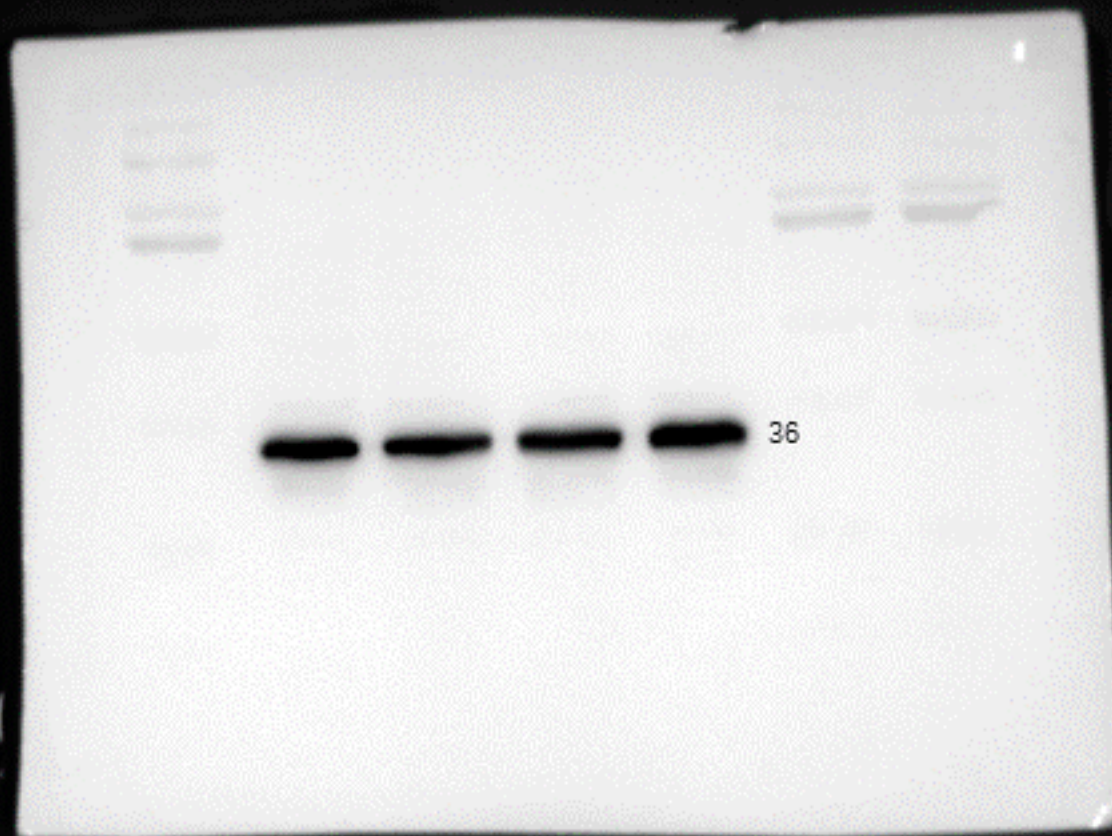

KDA

70

55

40

36

GAPDH

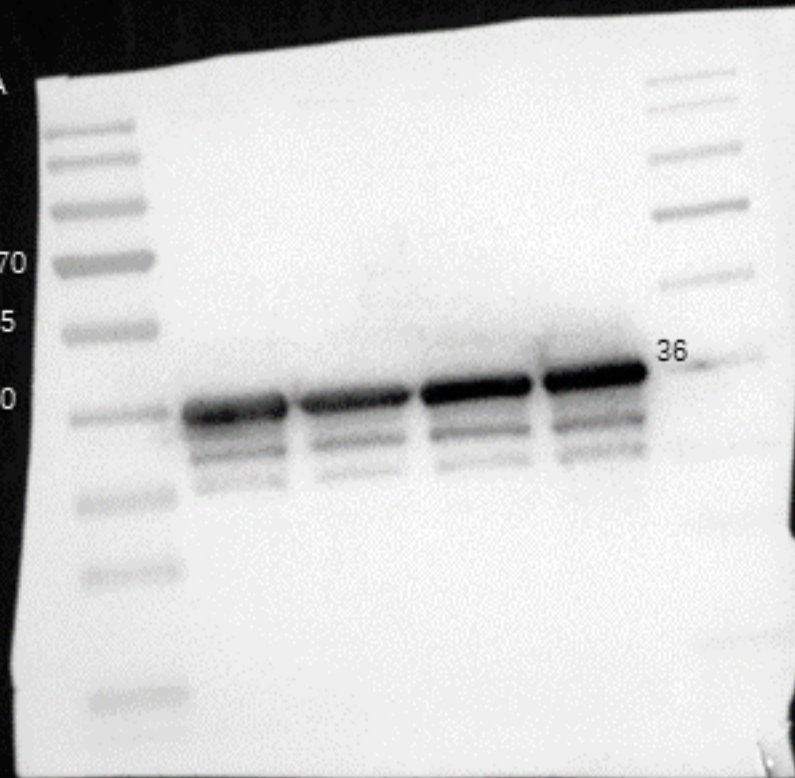

KDA

70

50

72

MMP2

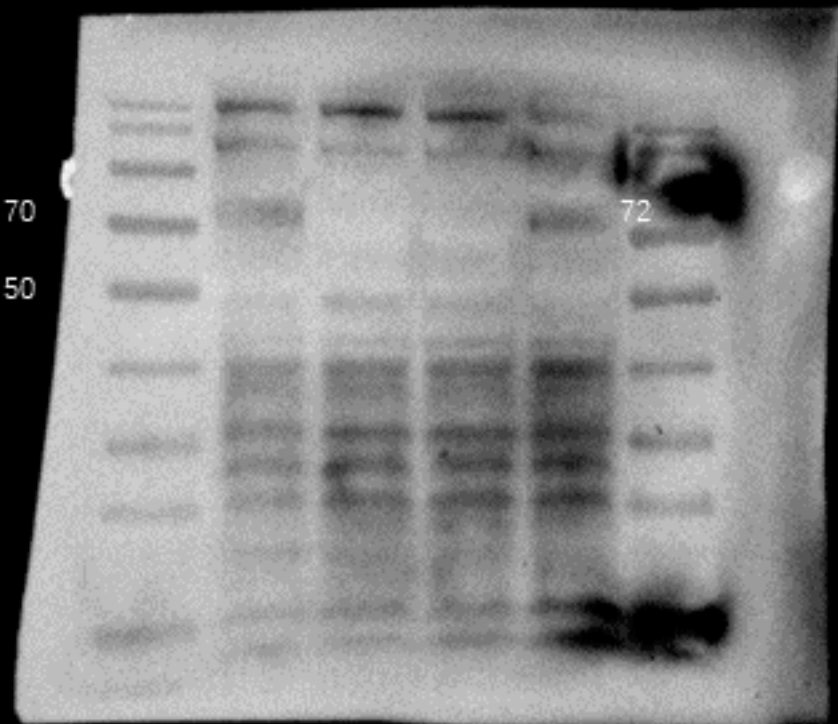

Supplement: Supplementary file 1 — Figure S1. [file CAM4-13-e70072-s001.pdf]
